# Physostigmine in Anticholinergic Poisoning: An Old Antidote With Resurgence

**DOI:** 10.7759/cureus.11739

**Published:** 2020-11-28

**Authors:** Nicholas G Blackstone, April Olson, Bujji Ainapurapu

**Affiliations:** 1 Internal Medicine, University of Arizona College of Medicine - Tucson, Tucson, USA

**Keywords:** physostigmine, anticholinergic delirium, anticholinergic overdose

## Abstract

Physostigmine is a cholinesterase inhibitor used therapeutically in patients with anticholinergic delirium that is so severe that intubation may be required for airway protection. Given that a wide variety of medications have anticholinergic properties, and the current standard of care is typically a central nervous system depressant, hospitalizations are often lengthy and normally require a critical-care level of attention. Despite this, physostigmine is often underutilized and poorly understood in the clinical setting. We report a case of a 43-year-old female who presented to the emergency department one hour after ingesting approximately 150 tablets of diphenhydramine in a suicide attempt. She was treated with benzodiazepines with minimal success, and airway protection became imminent as her mentation continued to decline. Through the use of physostigmine, we were able to save this patient from endotracheal intubation and the potential complications of mechanical ventilation.

## Introduction

The central role of physostigmine in anticholinergic delirium is to inhibit acetylcholinesterase. By inhibiting this enzyme, synaptic acetylcholine breakdown is reduced, thereby out-competing other molecules at the muscarinic receptors and inhibiting their actions. For this reason, physostigmine is exceptionally useful in treating anticholinergic delirium.

Anticholinergic delirium is a life-threatening clinical manifestation often requiring intubation and prolonged intensive care unit (ICU) stays. This is largely due to benzodiazepines being used as the primary treatment for severe delirium while also being associated with disinhibited delirium [1]. 

This case highlights the underutilized nature, safety, and efficacy of physostigmine in treating anticholinergic delirium and underscores the importance of considering physostigmine as a viable treatment option in patients with severe anticholinergic delirium.

## Case presentation

A 43-year-old female presented to the emergency department one hour after ingesting approximately 150 tablets of diphenhydramine. She was febrile to 38.2 degrees Celsius and tachycardic to the 150 beats per minute with a Glasgow Coma Scale (GCS) of 13. On physical exam, she was initially noted to be diaphoretic with psychomotor agitation but lacked mydriasis or notable skin dryness, findings suggestive of multi-drug overdose rather than a classic anticholinergic syndrome. Initial labs were normal except for elevated lactate to 3.8 millimoles per liter (mmol/L) with undetectable levels of salicylate and acetaminophen. The ethanol level was elevated to 39 milligrams per deciliter (mg/dL) and urine toxicology was positive for cannabis. An electrocardiogram (EKG) showed sinus tachycardia with a prolonged QTc. Despite an initial response to lorazepam, reexamination was concerning for a GCS of 9.

The medical intensive care unit (MICU) was consulted over concern for her inability to protect her airway. In an attempt to avoid intubation, a 2mg bolus over 4 minutes of physostigmine was trialed. Within minutes, her mentation began to improve. However, the patient's initial symptoms returned after the first dose wore off. The patient was subsequently started on a physostigmine infusion and was transferred to the MICU for further management.

In the MICU, she tolerated the physostigmine infusion at an initial rate of 0.5 mg/hr with an acceptable titration up to 1.0 mg/hr, as recommended during multidisciplinary rounds with toxicology, pharmacy, and the MICU team. The ultimate infusion-rate was dictated by clinical improvement in her mental status, given this was the patient's most prominent clinical feature. EKGs were repeated every two hours along with continuous cardiac monitoring to evaluate for the widening of the QRS and ventricular or bradyarrhythmias.

Her physostigmine infusion was discontinued five hours following admission to the MICU. Before discontinuation of the infusion, the patient's mental status was back to baseline, and the fever and tachycardia present on admission had resolved. On physical exam, the patient lacked any persistent or residual signs of anticholinergic toxicity. Her repeat labs were all within normal limits and she was transferred to the medical floors on hospital day two for continued management and discharge planning.

## Discussion

This case demonstrates that physostigmine can be used safely and effectively in preventing endotracheal intubation in a patient with anticholinergic drug-related severe delirium. In this article's following subsections, we will discuss the relative and absolute contraindications when using physostigmine, evaluate dosing strategies, explore why physostigmine fell out of favor, and understand it's safety profile and effects on hospital length of stay.

Absolute and relative contraindications

Although our patient had an apparent history of anticholinergic overdose, her symptomatology did not match her history at the time of presentation. We were very concerned administering physostigmine initially as one of the major safety concerns are in the setting of multi-drug overdoses, were both anticholinergic and cholinergic effects are in play (i.e., tricyclic antidepressant [TCA] overdose) and adding another cholinergic agent into the mix can lead to cholinergic toxicity. For this reason, according to the package insert, any known or suspected TCA overdose is an absolute contraindication to using physostigmine for treatment of severe delirium in this setting. Additionally, caution should be used in patients who have ingested a known proconvulsant drug because excessive cholinergic stimulation can affect the central nervous system (CNS) and decrease the patient's seizure threshold [1]. additional absolute contraindications include reactive airway disease, intraventricular conduction defects and atrioventricular (AV) block, intestinal and bladder obstruction, and peripheral vascular disease.

Why did physostigmine fall out of favor?

A case series in the 1980s described two cases in which physostigmine was used to treat the anticholinergic effects of TCA overdose that resulted in asystole [2]. With respect to TCAs, asystole was mostly secondary to the cardiotoxic effects seen in cholinergic toxicity. We now know that this is an absolute contraindication and having continuous cardiac monitoring and atropine at the bedside largely mitigates the risk of adverse events.

Dosing strategies

Different dosing strategies exist in the medical literature. In our case, we trialed a 2mg bolus over four minutes. Subsequently, we initiated a continuous infusion at a rate of 0.5 mg/hr with an acceptable titration rate of up to 1.0 mg/hr. The continuous infusion was safely titrated down at a rate directly proportional to her mentation. Some literature recommends not starting a continuous infusion at all but instead giving an initial bolus followed by 0.5 to 1.0 mg every five minutes until delirium has resolved or cholinergic signs appear [1]. Either approach appears to be acceptable, and patients should be on continuous cardiac monitoring and atropine must be available at bedside.

Physostigmine vs. benzodiazepines

Our case demonstrates how the sedative effects of benzodiazepines can be ineffective and may further suppress the patient's neurological ability to protect their airway. In a study conducted in 2000, physostigmine reversed agitation and delirium in 96% and 87% of patients, respectively. Benzodiazepines controlled agitation in 24% of patients but were ineffective in reversing delirium [3].

Safety profile

A 10-year retrospective cohort study was conducted in 2018 showed physostigmine to have a good safety profile with the ability to improve, and in some cases, resolved anticholinergic delirium when administered in doses less than 2mg [4]. These finding are further supported by a meta-analysis published in 2019 which analyzed data of 161 papers, and 2,299 patients concluding that physostigmine was a safe antidote to treat an anticholinergic overdose [5].

Hospital length of stay

Without the need for endotracheal intubation, our patient's length of hospital stay was drastically reduced. A study conducted in 2010 evaluating physostigmine use in anticholinergic delirium and hospital stay showed that 42% of patients were discharged directly from the emergency department, 31% were admitted to the general medical floors, and 27% were admitted to the intensive care unit with an average length of stay being 3.59 days [6]. A more recent retrospective study conducted in 2019 evaluated 141 patients in whom physostigmine was used demonstrated reduced intubation rates without increased incidence of dangerous adverse events including bradycardia and seizures. The study further elucidated that this in turn minimizes critical care time and thus decreases resource utilization in the management of an otherwise complicated patient population [7].

## Conclusions

This case illustrates the importance of early physostigmine use in the setting of an anticholinergic overdose. The current literature supports using physostigmine with caution while being aware of the contraindications. The rapid administration of physostigmine in a patient with a moderate pretest probability of anticholinergic toxicity allowed us to avoid intubation and a potentially protracted hospital course. Despite the atypical nature of her initial presentation, this case demonstrates the clinical utility of physostigmine and its use in such cases. The benefits of physostigmine administration outweigh the risks of intubation and may decrease morbidity, mortality, hospital stay, and healthcare costs.
